# Vestiges of the Natural History of Creation

**Published:** 1845-10

**Authors:** 


### REVIEWS.

---

ART. VII.—*Vestiges of the Natural History of Creation.* Second edition, from the third London edition, greatly amended by the author; and an Introduction, by Rev. GEORGE B CHEEVER, D. D. New York: Wiley & Putnam. 1845. 12mo. pp. 280.

[CONCLUDED.]

In our last number, after a pretty full exposition of the doctrines of this singular work, we proceeded to state what to our minds is a fatal objection to its leading hypothesis; namely, that it is unsupported by facts. We see nothing in the living world that favors it, and unless we renounce observation, and all inductive reasoning, we must discard it as one of the fancies in which philosophers of the Monboddo, Darwin, and Lamarck school have loved to indulge. If it was by a transmutation of species that the world was peopled with its present inhabitants, how happens it that we see now no such changes going on among its countless tribes? Nature rebels against such innovations and admixtures. The horse and the ass leap her barriers once, as do some of the domestic fowls, the cock and guinea hen, and certain varieties of ducks, for example; but with a single cross, productiveness ceases; sterility is stamped upon their issue. And to the races more widely separated even this latitude is not allowed. It is not believed, by those who have examined the subject most carefully,* that the dog will cohabit with the

---

* See this Journal, vol. I, (new series) p. 514, for some very striking facts bearing on this subject.

fox or the wolf, closely as the species are allied; and mixed breeds of canine and feline animals, or of ruminants and pachyderms, no one expects to see, any more than the mermaids and minotaurs of ancient fable.

There is another objection, which is pressed by Dr. Cheever, in the introduction, with great effect, and which must occur on a little reflection to every mind. It is the incapacity of monkeys to rear human infants. The argument is conclusive; it needs but to be presented to carry conviction to every understanding not blinded by hopeless prejudice. No one will believe that helpless new-born babes could be nurtured on monkey-parents' knees, and it is strange that the absurdity of such a conclusion did not drive the ingenious author from a system which leads to it.

Should this doctrine of the progressive development of organic life prove, after mature investigation, to rest upon the sure basis of fact, there will be no alternative left but to embrace it, nor are we able to perceive any good reason why we should meet it with fixed hostility. If it be founded in nature, most surely we are safe in receiving it, for nothing is more true than the saying, that nature knows no useless or dangerous truth. If the fossilized remains of plants and animals do incontestably show a gradual development of living things, let us accredit them as the veritable handwriting of the Almighty, and rest satisfied that it was by such steps that he proceeded in the work of creation. But shall we also adopt the doctrine of spontaneous generation, and conclude that the species have come one from another, beginning with polypi, and running "from harmony to harmony," as the poet has it, "the diapason closing full on man?" Not at all. Take it, that the lowest orders existed first, that to them succeed species a little higher in the scale of being, the present perfect races appearing last, and we have only an expression of the fact that in this order, for reasons unquestionably wise, although they may not be wholly apparent to us, they were spoken into being.

But do these medals of creation, as they have been happily styled, afford conclusive proof of such a law of development? They do not, in the judgment of some of the most learned men of the age who have given their minds to the question. Mr. Lyell, one of the first, if not the very first of living geolögists, after a careful examination of the whole ground, has expressed his opinion that the doctrine "has but a slender foundation in fact." In matters of this sort authority must not be strained too far, but certainly the conviction of a man who has attentively considered all the facts in the case, is entitled to a good deal of weight. We will see what some of the facts are which are thought to militate against this doctrine.

We referred incidentally to one of these in the former part of this article, in which we mentioned the appearance of birds at so early a period as that of the new red sandstone. The epoch when they left their foot-prints in the sand-stone of the Connecticut, is more ancient than those of the European formations from which their most extraordinary extinct reptiles have been obtained, and we should hence be led to infer that birds were created before lizards, the law of development being reversed, and the higher appearing earlier than the lower. This anomaly is met by the advocates of the law of development by objecting, that these birds belonged to a very imperfect family of birds, destitute of wings, and with only a rudimentary sort of respiratory apparatus; in fact, hardly superior to reptiles, and as well adapted as they to the poor accommodations which mother earth afforded to her first inhabitants. If size could be urged as an argument for high organization, some of these extinct cassowaries might fairly lay claim to it, for the tracks to which we have referred prove that some of them had a stride of six feet. Their stature, judging from their stride, and the length of their feet, no less than nineteen inches, was nearly two-fold that of the African ostrich, and in their rambles along the margin of the early sea, from which this sand-stone was deposited, they appear to have been accompanied by more than twenty species of smaller birds. Who will undertake to say that all of these were incapable of flight, having only rudiments of wings and lungs? But if they were fitted for flying, then they must have required an atmosphere constituted like the present, and this is opposed to the doctrine of progressive development.

The following facts are equally hostile to it, for which we are indebted to Mr. Lyell, who opposes the doctrine with a strong array of arguments. It is not only the remains of polypi, and of such humble races of animals, that we find in the early formations, but the bones and skeletons of fishes. Geologists have discovered these in the old red sand-stones, and even in some transition lime-stones; in other words, they have found vertebrated animals in the most ancient strata, concerning the fossils of which we are in possession of any accurate information.

When we come to the tertiary formations, in which the organic remains of quadrupeds abound, we find proofs enough of a succession of races, but no evidences of progressive development. In the Eocene, or oldest strata of that class, the remains of a great assemblage of mammiferous animals have been brought to light, all of the extinct species, but apparently of an organization as perfect as that of any of the existing races. In the Miocene beds, or those of a newer tertiary epoch, other forms have been discovered, for the most part also of lost species, and almost entirely distinct from the Eocene tribes, but in no respect an improvement upon them. And, in like manner, with the Pliocene, or later period, we have a change in fossils, but no development. In the succession of quadrupeds through these long ages we detect no signs of progress in organization—no indication that the fauna of the earliest period was less perfect, in form or function, than that which immediately preceded our own.

If we turn now to the vegetable kingdom we shall find that the facts are equally unfriendly to this hypothesis. Instead of a few of the simplest forms of vegetation, we come at once, in the rocks which retain the traces of plants, upon all the leading divisions of the vegetable kingdom. We discover not the slightest indication of a gradual advance from the cryptogamous, up to what are usually called the higher order of plants; but, on the contrary, among the first three or four hundred species brought to light, have been discovered some of the more fully developed forms, both of dicotyledons and monocotyledons.

We have said that the author of the Vestiges is a believer in spontaneous generation, and this doctrine, indeed, would seem to be necessary to his hypothesis of a progressive development. It may be that the use which he makes of this exploded fiction is one cause of the aversion with which so many regard his system. We say exploded, for if any notion in physiology can be regarded as obsolete, we think the idea of spontaneous generation ought to be. It has been so often driven from its ground, as the light of science has widened, that one wonders that it has not long since ceased to command the respect of scientific men. While insects and cryptogamous plants perpetuated the species by processes which had not been discovered, their generation was held to be spontaneous; but when, at length, it was ascertained that, in analogy with the superior orders, they propagated by eggs and seeds, the doctrine took refuge in the infusory animalcules. Driven finally by the microscope from this strong hold, the last hope of its advocates is in the insects brought to light by the galvanic experiments of Mr. Crosse. How long this frail shelter will give the semblance of protection to the doctrine remains to be seen, but, we are persuaded, not long.

We are a little too fast. Says the author of the Vestiges:

"When lime is laid down upon a piece of waste moss ground, and a crop of white clover for which no seeds were sown is the consequence, the common explanation is, that the seeds have been dormant there for an unknown time, and were stimulated into germination when the lime produced the appropriate circumstances. How is it possible to be satisfied with this hypothesis, when we know (as in an authentic case under my notice) that the spot is many miles from where clover is cultivated, and there is nothing for six feet below but peat moss, clover seeds being, moreover, known to be too heavy to be transported, as many other seeds are, by the winds?"

We read, a short time since, a similar case, and of unquestionable authenticity, in an agricultural paper. In making an excavation for a road, or for some such purpose, a quantity of the clay from the side of a hill was spread over the ground a little way off, and the next season this newly-made soil was covered with white clover. The circumstance was thought remarkable as the earth was taken from a considerable depth; but some one, curious in the matter, solved the enigma by finding, on a close examination, an abundance of clover-seed in the hill from which the clay was taken.

Another authentic case occurred in this city a few weeks ago. From an isolated pond in the neighborhood some boys brought us a muscle with which we were unacquainted. We showed it to our friend, Mr. Beadle, who has a familiar acquaintance with the shells of the Ohio, and for a time he was at a loss what name to assign it; but at last he decided that it was the *Unio obesus*, a species which up to that time had not been found in this locality. The pond on further exploration proved to abound in this species, and in it alone. How came they there? The explanation of those who hold to the doctrine of invariable generation, of course, would be, that either the muscles or their eggs, at some former time, were transported thither. "But how," would retort our author, "is it possible to be satisfied with this hypothesis," seeing that the spot is many miles from where this species has been found in the river? Well, since we discovered this Unio in its secluded home, Dr. Shumard has met with it in the Beargrass, a mile or two distant; and now, whether its transportation to this pond by some of the many agencies that offer in such cases, or its generation there be the more probable hypothesis, is a question we cheerfully submit to the good sense of the reader. The animal proves to be the U. Sayi.

We have not the leisure, and perhaps our readers think could not well afford the space, to discuss this question at greater length, and with a remark or two more, we shall therefore bring this notice to a close. Since beginning our article we have learned that several spirited and able reviews of the Vestiges have appeared, in which the speculations of the author are keenly criticised, and as the respectability of the sources from which these notices emanate will secure them a full share of public attention, we deem it of the less consequence to push our objections further. We are glad to find that journals of so much influence as the Edinburgh, and the North American Review, have taken the work in hand, and that the antidote is thus likely to circulate as extensively as the poison.

Having indulged our strictures so freely on the doctrines of this work, we may be permitted to say, in conclusion, that it is not so bad a book as the preface of the American editor prepared us to find it. It does not teach atheism, but in a passage towards the close even affords what we conceive to be a strong argument for the Christian religion. The author had been dilating on the mental constitution of animals, and on the possession by man of faculties which connect him "with the things that are not of this world"—as veneration "prompting him to the worship of the Diety," and hope "to carry him on in thought beyond the bounds of time;" with reason, "to enable him to inquire into the character of the Great Father, and the relation of us, his humble creatures, towards him." The existence of faculties having a regard to such things he holds to be good evidence, "that such things exist." Mental science goes thus far in support of religion, but no farther, and for the rest, he adds, we must look to evidence of a different kind. That evidence it was not the design of his book to indicate or examine; but in favor of its existence he closes his volume with the following impressive thoughts:

"The history and constitution of the world have now been explained according to the best lights which an humble individual has found within the reach of his perceptive and reasonsoning faculties. We have seen a system in which all is regularity and order, and all flows from and is obedient to a divine code of laws of unbending operation. We are to understand from what has been laid before us, that man, with his varied mental powers and impulses, is a natural problem, of which the elements can be taken cognizance of by science, and that all the secular destinies of our race, from generation to generation, are but evolutions from a primeval arrangement in the counsels of Diety. It does not, according to this view, appear necessary that God should exercise an immediately superintending power over the mundane economy; he might be pronounced to repose in silent contemplation of his works, unoffended by evil, pitiless of suffering, satisfied with one eternal round of such doings as we see exemplified upon earth, liable as these presumably are to a progress in an improving direction. But this view, however supported, being attended with these sequences, is certainly one which no large portion of mankind will ever embrace. It may be a view of truth, but there is a monitor within which denies that it is the whole truth. We intuitively shrink from it in its isolated sternness, and demand to know if there are not other truths which require to be associated with it before it can be received even in its most limited application.

"To such requirements of our nature, so that we are satisfied of their being purely intuitive, and so I consider the present to be—it is necessary that the philosopher give full attention, for they are as truly facts as any other which he ever has occasion to consider. Such instinctive apprehensions cannot be there for nothing, for no such thing is made in vain. Reasonings may appear to be against them, and for ages they may be destitute of that kind of proof which rigid seekers for truth demand. But how often has it happened that they have after all been shown and admitted as true! Forty years ago—to take an example—it was advanced by one philosopher, and approved by many, that population tends to advance more rapidly than the productive powers of the soil, so that many human beings must come into the world, only by an irreversible doom of nature to sink out of it again. The notion was repelled by mankind generally, as disrespectful to Providence, and suggesting a painful idea of the constitution of human nature. For years the objection was thought by the disciples of Mr. Malthus to be futile; but its validity is now pretty generally acknowledged by the men of highest intelligence. It is seen that the philosopher erred in his calculations, and was therefore wrong in his conclusions. The lowly and unpretending minds are allowed to have been, albeit on no ratiocinative grounds, in the right. It was in considering such triumphs of unenlightened judgment, that Pascal gave forth his beautful saying, that the heart has its aphorisms as well as the understanding. Such impulses appear to be the fore-cast shadows of great truths, and, when they are clearly seen to spring from no superficial or evanescent feeling, are assuredly worthy of being taken into account in all questions to which they relate.

"So thinking, I would seek to add to the truths which have already been eliminated from facts ascertained in science, some others which claim a place on the strength of their being dictated by the universal feelings of man. Something in our nature—as it appears to me—tells us that the Author of the universe is nearer to us, is in a more familiar and paternal relation to us, than would seem to be implied by a theory which represents him as only an author of laws. We cling to the idea that He continually watches over us, that we can come by rightly directed thought into communion with him, and that, when life's changeful scene is over, we shall, if found worthy, be received in a new form of being into his fatherly bosom. We feel, in our dependent state here below, a need for some ultra-mundane being, on whom to rest, as we would do upon the breast of a friend, and to whom to look as an ultimate refuge from the trying vicissitudes of life. We also feel how far short our best doings and designings are of that perfect goodness which our imagination can suppose—how deeply injurious and offensive must our ordinary life be to one so purely good. Something seems necessary to reconcile us to him, or to fit us for being restored to his society. Hence the idea of penitence and its wondrous potency—hence, in short, religion. Now these emotions are all so natural to man, they rise so readily in the civilized bosom, and meet so ready a reception in all neophytes who have not been perverted by baser feelings or grossly corrupt systems, that, if the principle which has been explained be a right one, they must point to truths. Admit that our reason cannot at present entirely justify them, we may expect that it will yet do so. They may be regarded (putting all other evidence aside) as truths in the dawning stage—suggested by the feelings—waiting only the final approbatory stamp of the understanding, and sure in time to receive it."

These emotions are met and satisfied by the Bible. In it is revealed that "system of mercy and grace" hinted at by our author, which comes up to all the aspirations of the human heart. It teaches fully, what he finds it "necessary to suppose," namely, "that the present system is but a part of a whole, a stage of a Great Progress, and that the Redress is in reserve." We are most unwise, if, while listening to the teachings of nature, we live heedless of its warnings.

*Note.*—In reviewing what we have written in respect to spontaneous generation, it has occurred to us that we have used language a little more positive than the present state of our knowledge on the subject would authorise. As this is one of the errors of which we have accused the author of the Vestiges, we are anxious to avoid it, and to assert nothing as fact which is not fully authenticated. It, perhaps, cannot be said that the microscope has *settled the question*, as to the mode in which the infusory animalcules propagate their species; it may be that the ova of beings so diminutive as some of them are—less than the twelve-thousandth part of an inch in diamter—are too small to be detected by the most powerful instruments; but we cannot help believing that what has been revealed by the microscope is sufficient to show, that the generative act in them is in perfect analogy with that function in all the higher races of animals. That such is the opinion of Professor Owen, the highest authority on the subject, will be seen by the following extract from his Lectures:

"The act of of oviparous generation, that sending forth of countless ova through the fatal laceration or dissolution of the parent's body, is most commonly observed in the well-fed *Polygastria*, which crowd together as their little ocean evaporates; and thus each leaves, by the act of its life, the means of perpetuating and diffusing its species by thousands of fertile germs. When the once thickly tenanted pool is dried up, and its bottom converted into a layer of dust, these inconceivably minute ova will be raised with the dust by the first puff of wind, diffused through the atmosphere, and may there remain long suspended; forming perhaps their share of the particles which we see flickering in the sunbeam, ready to fall into any collection of water, beaten down by every summer shower by the streams or pools which receive or may be formed by such showers, and by virtue of their tenacity of life, ready to develope themselves wherever they may find the requisite conditions for their existence.

"The possibility, or, rather, the high probability, that such is the design of the oviparous generation of the *Infusoria*, and such the common mode of the diffusion of their ova, renders the hypothesis of equivocal generation, which has been so frequently invoked to explain their origin in new-formed natural or artificial infusions, quite gratuitous. If organs of generation might, at first sight, seem superfluous in creatures propagating their kind by gemmation and spontaneous fission, equivocal generation is surely still less required to explain the origin of the beings so richly provided with the ordinary and recognized modes of propagation."

We have neither space nor leisure to pursue this subject, but an experiment which we find reported by Prof. Owen is of so conclusive a character that we make room for a notice of it. Prof. Shulze, of Berlin, filled a glass flask half full of distilled water, in which were various animal and vegetable substances; he then caused the water to boil violently, and while the vapor was escaping adapted bent tubes to the flask, one containing concentrated sulphuric acid, and the other a solution of potash, by means of which he could draw atmospherical air through the water, at the same time that all access to it, except through the corrosive fluids, was effectually cut off. The boiling heat, it was conjectured, would kill every living thing, and all germs in the flask, while the passage of the air through the acid and potash would be likely to have the same effect. For more than two months it was exposed to the influence of summer light and heat, the air being changed daily by suction through the tubes; during this time the experimenter watched with a microscope, but discovered in the water no "living animal or vegetable substance;" and when at length he separated the different parts of his apparatus not the slighest trace of "infusoria or confervæ, or of mould, could he find in the whole liquid; but all three presented themselves in great abundance a few days after he had left the flask standing open." A vessel with boiled water placed near the apparatus and exposed to the air was found to contain infusoria in a day or two.

